# Plastic pollution and health

**DOI:** 10.1016/j.eclinm.2023.102074

**Published:** 2023-06-22

**Authors:** eClinicalMedicine

June 5 marks World Environment Day, which is now in its 50th year. The theme for 2023 is plastic pollution, under the campaign #BeatPlasticPollution. Despite efforts in recent decades to curtail the overwhelming flow of plastics, the scale of plastic production has surpassed every other material since the 1970s and the majority of these products are destined for landfill sites or leak into the environment. Perhaps most damning of all, as much as 50% of all plastic products produced (380 million tonnes annually) are only used once, and only 9% of plastics are recycled globally. Although the effects of plastic pollution on our climate, wildlife, ecosystems, and the economy are vast and often highlighted, the effect on human health should not be underestimated.

Microplastics—which are plastic fragments smaller than 5 mm that are released into the environment during the abrasion or breakage of plastic objects—can enter the human body through ingestion, inhalation, and even dermal absorption, and accumulate in organs. A subset of microplastics (<1 μm in length) are often referred to as nanoplastics. WWF estimations suggest that humans may ingest up to 5 g of microplastics weekly. To date, microplastics have been detected in the blood, breastmilk, placenta, and lower airway of humans, to name just a few. This is not surprising considering that these plastic fibres have infiltrated the drinking water of cities and towns all over the world, with the USA, Lebanon, and India having the highest prevalence in samples collected in 2017. The possible routes for water contamination are numerous. One simple example of the route these particles could take is from textiles, to washing machine effluent, to wastewater treatment facilities, to waterways and groundwater, to water treatment facilities, to potable tap water. As well as directly drinking such water, plastics now occupy our food chain—with these pollutants detected in meat and cow's milk (including beef, pork, and their feed), fish and seafood, table salt, and fruit and vegetables.

Despite this being an early area of research, the presence of microplastics has been linked to respiratory complications, endocrine disruption, inflammatory bowel disease symptom severity, narrowing of fetal capillaries, and stomach and oesophageal cancers. Endocrine disruption is the most well studied of these risks, with the endocrine-disrupting chemicals found within plastics (eg, phthalates, perfluoroalkylsubstances, bisphenol A, and flame retardants) being linked to a myriad of health effects from endometriosis and breast cancer to heart disease and obesity. The underlying mechanisms for these potential effects via microplastics are, as yet, unclear. It also remains to be elucidated how long microplastics remain in the body before being excreted or exhaled.

In addition to microplastics, the health impacts of plastics are felt at every stage of the plastic lifecycle. As outlined in a recent report by the Center for International Environment Law (Plastic & Health: The Hidden Costs of a Plastic Planet) and The Minderoo-Monaco Commission on Plastics and Human Health, the occupational harms of plastic pollutants are vast and diverse. Specifically, individuals who extract fossil carbon feedstocks for plastic production suffer increased mortality from coal workers’ pneumoconiosis, silicosis, cardiovascular disease, chronic obstructive pulmonary disease, and lung cancer. Plastic production workers are at increased risk of leukaemia, lymphoma, hepatic angiosarcoma, brain cancer, breast cancer, mesothelioma, neurotoxic injury, and decreased fertility; whereas plastic recycling workers have increased rates of cardiovascular disease, toxic metal poisoning, neuropathy, and lung cancer. These disproportionate harms should not be ignored, and additional safety legislation is required to ensure that workers are sufficiently protected.

The solutions to the plastic crisis are, unsurprisingly, not simple. Although recycling has a key part to play, evidence has shown that this process might be making the microplastics problem worse. Moreover, current recycling systems cannot handle the huge global volume of plastic produced and usually rely on the export of plastic waste from high-income to low-income countries—with illegal exports and dumpsites now widespread. Even so-called biodegradable plastics and textiles are not the saviour they might seem to be, given that they often require high temperatures for breakdown and can disrupt ocean life.

Although various innovative solutions have been proposed—such as bioplastics, refill stations, and artificial intelligence-based recycling facilities—and they undoubtedly have value, the best way to keep plastic out of our air, water, and soil is to drastically cut how much we use and revolutionise its design and disposal. The circular economy model is the most convincing solution proposed thus far. According to a new report by the UN Environment Programme, plastic pollution could be reduced by 80% by 2040 if nations, governments, and companies make extensive policy changes and market shifts. After eliminating unnecessary plastics to reduce the size of the crisis, these shifts center on reusing, recycling, and reorienting and diversifying products. As well as economic gains of US$4.5 trillion, the UN report suggests these radical changes could also result in a net increase of 700,000 jobs by 2040, mostly in low-income countries, substantially improving the livelihoods of millions of workers. A key feature of the circular economy is that manufacturers are legally and financially responsible for the safety and disposal of all materials they produce and sell, so policy changes are likely to have a huge impact.

This systemic change is what is needed to stop the flow of plastic and its byproducts. Such a lifecycle approach to plastics would revolutionise everything from extraction of raw resources to product design, production, consumption, and waste management. If historic growth trends continue, worldwide production of primary plastic is predicted to reach 1100 million tonnes by 2050. Decisive action needs to be taken by governments globally to ensure this is not the case and we as individuals need to take responsibility for decreasing how much plastic we use. More research is needed into the harms that plastic pollutants pose to humans, but we know enough to see that change is needed. Urgently.

